# Varicella outbreak at the Itamaraju Indigenous Health Base Center: a descriptive study, Bahia, 2024

**DOI:** 10.1590/S2237-96222026v35e20250856.en

**Published:** 2026-02-13

**Authors:** Guilherme Alves de Siqueira, Leticia Stanczyk, Benvindo Joãozinho Sá, Lucimeire Neris Sevilha da Silva Campos, Vanessa Cristina Fragoso Farias, Pedro Ricardo da Silva Biscarde, Ana Carolina de Castro Silva, Aluciena Dias da Silva Dias, Sonia Helena Afonso, Adriana Dourado de Carvalho, Rosidalva Barreto da Silva Sousa, Tatiane Ferreira dos Santos, Solange Nunes Souza Monte, Octavia Lorena P. Falcao, Samara Carolina Rodrigues, Aroldo José Borges Carneiro

**Affiliations:** 1 Ministério da Saúde, Secretaria de Vigilância em Saúde e Ambiente, Brasília, DF, Brazil; 2 Distrito Sanitário Especial Indígena da Bahia, Salvador, BA, Brazil; 3 Distrito Sanitário Especial Indígena da Bahia, Itamaraju, BA, Brazil; 4 Secretaria da Saúde do Estado da Bahia, Salvador, BA, Brazil; 5 Secretaria da Saúde do Estado da Bahia, Teixeira de Freitas, BA, Brazil; 6 Secretaria Municipal de Saúde de Prado, Prado, BA, Brazil

**Keywords:** Varicella, Disease Outbreak, Indigenous Population Health, Vaccination, Descriptive Study, Varicela, Brotes de Enfermedades, Salud de las Poblaciones Indígenas, Vacunación, Estudios Descriptivos

## Abstract

**Objective:**

To investigate varicella cases in the Itamaraju indigenous territory base center and calculate the varicella vaccination coverage in the Tawá village, Bahia, 2024.

**Methods:**

Descriptive study of case series and census vaccination survey in children from the Tawá village conducted between June 6 and July 3, 2024, on the secondary survey database released by the General Coordination for Immunopreventable Diseases. We used descriptive statistics with measures of absolute and relative frequency and central tendency and dispersion.

**Results:**

We identified 42 cases, equally distributed between the sexes, with 27 indigenous persons and 19 children aged 7-13 years, and 14 cases received no varicella vaccine. There were 23 cases in the Tawá, Mucujê, Canto da Mata and Corumbauzinho villages, and 25 cases were associated to a school in the Tawá village, with indigenous and non-indigenous students, with an attack rate of 6.2% (25/403). In another indigenous school (Mucujê village), the attack rate was 7.0% (3/43). For the cases with available information (n=31), the most frequent symptoms were exanthema (31/31), pruritus (24/31) and fever (21/31). The varicella vaccination coverage was 90.0% (54/60) for the 1st dose and 70.3% (26/37) for the 2nd dose. Sixteen children received varicella vaccine after the outbreak began: seven received the first dose and nine received the second dose. Immunoglobulin was used for one pregnant woman. The outbreak was controlled 101 days after the first case.

**Conclusion:**

The occurrence of the outbreak in the indigenous territory was confirmed. The varicella vaccination status in the Tawá village was below the target recommended by the National Immunization Program. Vaccine updating and active case search were implemented.

## Resumen

**Objetivo:**

Analizar casos de varicela en el territorio indígena de la base Itamaraju y calcular la cobertura de vacunación contra la varicela en el pueblo de Tawá, en Bahía (Brasil), 2024.

**Métodos:**

Estudio descriptivo, de serie de casos, y encuesta de vacunación censal a niños del pueblo Tawá realizada entre el 6 de junio y el 3 de julio de 2024, en la base de datos secundaria de la investigación puesta a disposición por la Coordinación General de Enfermedades Inmunoprevenibles. Se utilizaron estadísticas descriptivas con medidas de frecuencia absoluta y relativa, y tendencia central y dispersión.

**Resultados:**

Identificamos 42 casos, distribuidos equitativamente entre los sexos; 27 eran indígenas; y 19 niños de entre 7 y 13 años de edad; 14 casos no fueron vacunados contra la varicela. Veintitrés casos ocurrieron en los pueblos Tawá, Mucujê, Canto da Mata y Corumbauzinho, y 25 estaban vinculados a una escuela en Tawá, donde estudiaban indígenas y no indígenas, con una tasa de ataque del 6,2% (25/403). En otra escuela indígena (pueblo de Mucujê) la tasa de ataque fue del 7,0% (3/43). Los síntomas de los casos más frecuentes con información disponible (n=31) fueron exantema (31/31), prurito (24/31) y fiebre (21/31). La cobertura de vacunación contra la varicela fue de 1.ª dosis 90,0% (54/60) y 2.ª dosis 70,3% (26/37). Dieciséis niños recibieron la vacuna contra la varicela después de que comenzó el brote, siete recibieron la primera dosis y nueve la segunda. Una mujer embarazada usó inmunoglobulina. El brote se controló 101 días después del primer caso.

**Conclusión:**

Se confirmó la ocurrencia del brote en el territorio indígena. La situación de vacunación contra la varicela en el pueblo de Tawá estaba por debajo de lo recomendado por el Programa Nacional de Inmunización. Se implementó la actualización de vacunas y la búsqueda activa de casos.

**Ethical aspects**

**This research respected the ethical principles, having obtained the following approval data:**

Research Ethics Committee: National Research Ethics Committee

Opinion number: 7.640.452

Approval date: 16/6/2025

Certificate of Submission to Ethical Appraisal: 88056825.4.0000.0008

Informed consent: Exempted by the research ethics committee.

## Introduction

Varicella is a viral, febrile, acute and highly contagious disease described by the appearance of maculopapular exanthema with centripetal distribution (center to extremities), which becomes vesicular, evolving into pustules and scabs that are dry and non-infectious, in a period of three to seven days^1^. The main symptoms of the disease are polymorphic skin lesions, which can present the various evolutionary forms in the same region of the body, in conjunction with pruritus^2^. The evolution in children is usually non-severe and self-limiting. In adolescents and adults, the clinic is usually more exuberant. Subclinical infections are rare^1^. Reports on varicella outbreaks in indigenous populations in Brazil are scarce, which hinders operational parameters for control.

The etiological agent is the varicella-zoster virus, family Herpesviridae, subfamily Alphaherpesvirinae, genome type deoxyribonucleic acid, having as reservoir the human being. After first infection, the virus remains latent in the nervous system and can be reactivated in adulthood, being known as herpes zoster^1^. It is transmitted directly from person to person (direct contact), through respiratory secretions (aerosols) or through the exudate of lesions (less frequent), and indirectly through contaminated objects^1^.

The disease has an incubation period of 10-21 days after contact with a varicella case. The transmissibility period is initiated two days before the onset of exanthema and lasts until all lesions are in the scab phase.

The disease is prevented through a vaccine, which is licensed in Brazil in monovalent or tetraviral presentations (measles, mumps, rubella and varicella components), made available by public health care services in their routine operation with a two-dose schedule: one dose at the age of 15 months and another dose at the age of 4 years. Children not vaccinated in a timely manner can receive the monovalent vaccine up to the age of 6 years, 11 months and 29 days^3^. Human antivaricella immunoglobulin is recommended as a prophylactic measure when the vaccine is contraindicated for specific groups, such as: children aged under nine months, pregnant women and immunocompromised people.

In outbreak situations, vaccination should be implemented selectively and as indicated in the National Vaccination CalendaR^3^, which recommends an opportune period of 120 hours (five days) for administration of vaccine and 96 hours (four days) for administration of human antiviral immunoglobulin, after contact with a suspected or confirmed case of varicella. Outbreaks and severe cases (hospitalized or deaths) are subject to immediate national compulsory notification. However, in the state of Bahia, Brazil, mild cases of varicella are also subject to compulsory notification, by completing the individual notification form of the Notifiable Disease Information System.

Clinical diagnosis is the main criterion for case confirmation, considering the symptomatology. Laboratory diagnosis is not used in the routine of the Public Health Care Laboratories network, except in the differential diagnosis of severe cases and deaths, or in less typical clinical presentations. Differential diagnosis can be made for other skin infections, such as: smallpox (eradicated); coxsackiosis; dermatitis herpetiformis; impetigo; Kaposi’s varicelliform rash; rickettsiosis, among others.

Searches in the literature found no reports on varicella outbreaks in indigenous population. However, there is a record of a varicella outbreak in daycare centers and schools in 2005, in the state of São Paulo, with 118 cases, one hospitalization and no death^4^ and another outbreak among Venezuelan immigrants housed in shelters in the state of Roraima in 2019, when 38 active cases were detected, one case presenting complications, with immunization actions taken to control transmission^5^.

On Mar 10, 2024, an 11-year-old indigenous patient from the Tawá village, Itamaraju Base Center, was treated by the Multidisciplinary Indigenous Health Care Team with fever, indisposition and presence of a gallbladder in the body, receiving a diagnosis of bullous impetigo. On April 13, 2024, with worsened clinical condition, the patient was taken by the guardians for medical care at the Prado Emergency Care Unit, with diagnostic confirmation of the first case of varicella in the Tawá village.

On May 14, 2024, seven cases had been notified, including a teacher from the indigenous school where the first case was a student, which was attended by indigenous and non-indigenous people. On May 17, 2024, the Multidisciplinary Indigenous Health Care Team reported the outbreak to the Special Indigenous Health District of Bahia. At that time, the varicella vaccination status in the Tawá village was unknown.

On May 29, 2024, the Special Indigenous Health District of Bahia invited the Training Program in Epidemiology Applied to the Services of the Unified Health System (EpiSUS-Advanced), together with the General Coordination for Surveillance of Immunopreventable Diseases, to support the investigation of varicella cases in the Tawá village.

On Jun 6, 2024, the EpiSUS-Advanced team and technical area of the General Coordination for Surveillance of Immunopreventable Diseases moved to the territory, with the arrival meeting held the next day. On Jun 10, 2024, in view of the possibility of more cases occurring in other villages of the Itamaraju Base Center, the Special Indigenous Health District of Bahia requested the expansion of the investigation site to the entire area under the scope of the Center.

The objectives of the study were to investigate varicella cases in the indigenous territory of the Itamaraju Center and to calculate varicella vaccination coverage in the Tawá village, Bahia, 2024.

## Methods

### Design

This is a study composed of two items: (i) description of varicella cases (case series) and (ii) census vaccination survey in children from the Tawá village, Itamaraju Base Center, extreme south of the state of Bahia, Northeast, Brazil.

### Background

The Itamaraju Base Center, according to demographic data from the Special Indigenous Health District of Bahia, had, in May 2024, 3,750 indigenous people of the Pataxó and Pataxó Hã Hã Hãe ethnic groups, divided into 22 villages distributed in the municipalities of Itamaraju, Prado, Alcobaça and a portion of Porto Seguro^6^. Notably, these villages include Tawá (410 inhabitants), Corumbauzinho (360 inhabitants), Mucujê (108 inhabitants) and Canto da Mata (95 inhabitants). This indigenous population has had contact with non-indigenous people since the 16th century and speak Portuguese. This region has intense tourist flow, especially the Corumbau district, in Prado^7^ ^), (^ ^8^.

### Participants

For the case series, the study population consisted of reported varicella cases between January 1, 2024 and July 3, 2024, with their respective contacts, identified through interviews with the cases or their guardians, in the area under the scope of the Itamaraju Indigenous Health Base Center. The following definitions were adopted:

- Suspected case of varicella: individual residing in the municipalities of the area covered by the Itamaraju Base Center, Bahia, who, from Jan 1, 2024 to Jul 3, 2024 was notified after presenting a mild condition of moderate fever, of sudden onset, which lasted two to three days, and generalized nonspecific symptoms (malaise, adynamia, anorexia, headache and others) and papulo-vesicular rash, beginning on the face, scalp or trunk (centripetal-head and trunk distribution);
- Confirmed case of varicella: suspected case with diffuse maculopapulovesicular exanthema whose vesicles evolved into scabs in two to three days, with no other apparent cause, with or without laboratory confirmation;
- Ruled-out case of varicella: suspected case whose clinical-epidemiological assessment is concluded as another disease;
- Contact: individual who had contact with a confirmed case within two days before the appearance of the rash until all lesions become scabbed;
- Varicella outbreak: occurrence of aggregate cases in daycare centers, schools, long-stay institutions, population deprived of freedom, indigenous areas, among others^9^. Varicella outbreaks are considered controlled after 21 days without new cases.

For the survey, the population consisted of guardians of indigenous people under the age of 7 years, living in the Tawá village, between Jun 6, 2024 and Jul 4, 2024.

### Variables of interest

The variables of interest related to the cases included sociodemographic characteristics (sex, age in years, race/skin color and place of residence) and clinical-epidemiological characteristics (case classification as confirmed or ruled out, clinical evolution, confirmation criteria, symptoms presented, dates of onset of symptoms, notification and clinical evolution, vaccination history, exposure history, and likely place of infection). For the contacts, we considered the same sociodemographic and clinical-epidemiological variables (dates of first and last contact with the case), nature of exposure (family, work relationship, school) and presentation of signs or symptoms in the last 30 days (Yes or No). The variables of interest related to the children participating in the survey were the verification of applied doses recommended for each age (respondent was asked if there were pending doses).

### Data sources

Anonymized secondary data released by the General Coordination for Surveillance of Immunopreventable Diseases, generated by the Training Program in Epidemiology Applied to the Services of the Unified Health System (EpiSUS-Advanced), during the investigation of a varicella outbreak. The notification data were obtained from the records of the Notifiable Disease Information System. Cartographic data were provided by the Special Indigenous Health District and public data were obtained from the Brazilian Institute of Geography and Statistics.

### Bias

Selection biases may have occurred, due to the possibility of underreporting of cases, and information bias, due to self-declaration. These biases were minimized through active searching and checking of records.

### Study size

Case series of 42 confirmed varicella notifications in the Notifiable Disease Information System, from the Itamaraju Indigenous Health Base Center. And census vaccination survey with 73 children living in Tawá village.

### Laboratory research

We performed serology to search for antibodies against varicella and reverse transcription followed by polymerase chain reaction (RT-qPCR) to search for Oropouche and Mayaro fever arboviruses, complementarily and not used as an inclusion criterion, only as a confirmatory or differential diagnosis.

The prevention and control initiatives implemented by the outbreak investigation teams were supported by indigenous health care teams, municipal and state immunization teams, with the administration of immunoglobulin in pregnant women and vaccination blockade and intensification, with tetraviral or monovalent vaccines, as per the recommendations of the National Immunization Program.

### Statistical analysis

Data analysis used descriptive statistics, with measures of absolute and relative frequency and measures of central tendency and dispersion. For cases in schools, where the exposed population was known, the attack rate was calculated using the equation:

$$\mathrm{Attack rate}=\frac{\mathrm{Confirmed cases of varicella}}{\mathrm{Exposed individuals}}*100\%$$

The incidence coefficient was also calculated for each village with recorded cases as follows:

$$\mathrm{Incidence coefficient}=\frac{\mathrm{Confirmed cases of varicella}}{\mathrm{Population of the village}}*100\;\mathrm{Inhabitants}$$

The incidence coefficient was used representatively to construct the thematic map.

The varicella vaccination coverage was calculated using the following equation:

$$\mathrm{Vaccination coverage}=\frac{\mathrm{Dose of vaccine applied to children}}{\mathrm{Eligible children per vaccine}}*100$$

We used the programs Go. Data 2.49.0, Microsoft Excel 2013, R.4.3.1 and R. Studio, QGIS 3.30.2 and Research Eletronic Data Capture (REDCap) 10.6.0.

## Results

There were reports of 57 suspected cases of varicella, with 42 confirmed cases and 15 ruled-out cases. Of these, five were diagnosed with allergy, four with impetigo, four did not evolve with varicella clinic, one with dermatitis and one with hand-foot-mouth disease. No deaths or severe cases of varicella were recorded. The vaccination survey covered 73 children, including the 69 indigenous children registered by the census of the Special Indigenous Health District in May 2024, adding four per change. We interviewed 61 guardians and all children had a vaccine record card.

**Figure 1:**
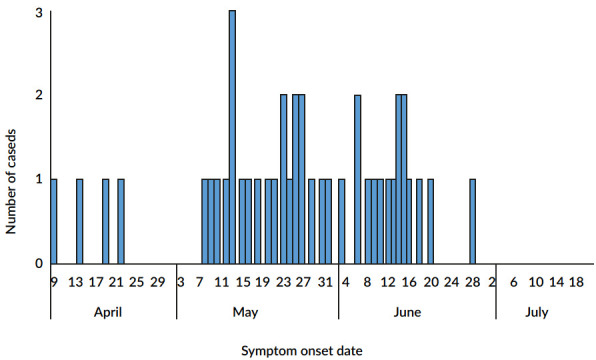
Confirmed cases of varicella, according to symptom onset date. Itamaraju Indigenous Health Base, Bahia, 2024 (n=42)

The first reported case had onset of symptoms on Apr 9, 2024. With active search, vaccination intensification and increased sensitivity of professionals, there was an increase in notifications, with a peak of records on May 13, 2024 (Figure 1). The last recorded case had a date of onset of symptoms on Jun 28, 2024. On Jul 19, 2024, after 21 days without a new case and 101 days since the first case, the outbreak was considered controlled (Figure 1).

In the 42 cases, there was an equal distribution between the sexes, 27 self-declared as indigenous, 13 as brown, one as white and one as black (Table 1). Twenty-three people reported living in indigenous territory, most of them in the Tawá village (n=16), followed by the Mucujê village (n=3), and the Corumbauzinho and Canto da Mata villages, with two cases each. Of the cases in non-indigenous territory, 18 resided in Corumbau, a district of the municipality of Prado 17 kilometers away from the Tawá village. When asked about the likely place of infection, of the 27 who were able to determine, 13 reported the school, 12 the residence and two the neighborhood as the likely place of infection (Table 1).

**Table 1:** Sociodemographic and epidemiological characteristics of the confirmed cases of varicella. Itamaraju Indigenous Health Base, Bahia, 2024 (n=42)

Variable	n
**Sex**	
Male	21
Female	21
**Race/skin color**	
Indigenous	27
Brown	13
White	1
Black	1
**Place of residence**	
Corumbau	18
Tawá Village	16
Mucujê Village	3
Canto da Mata Village	2
Corumbauzinho Village	2
Palmares	1
**Probable site of infection**	
School	13
Residence	12
Neighborhood	2
Unable to determine	15
**Age group (years)**	
1-7	13
8-14	19
15-21	5
22-28	1
29-35	2
>35	2
**Varicella vaccine**	
No	14
1 dose	13
2 doses	9
No information	6
**Signs and symptoms (n=31)** ^a^	**n (%)**
Exanthema	31 (100.0 %)
Pruritus	24 (77.4 %)
Fever	21 (67.7 %)
Headache	18 (58.1 %)
Lack of appetite	13 (41.9 %)
Irritability	8 (25.8 %)
Vomiting	3 (9.6 %)

^a^Information on signs and symptoms available only for 31 of the 42 cases.

The most affected age groups were 8 to 14 years (n=19) and 1 to 7 years (n=13). As for the varicella vaccination status, 14 of the cases were unvaccinated, followed by 13 people with one dose and nine people with two doses. Six people had no information on vaccination record. Signs and symptoms presented included exanthema (n=31), pruritus (n=24), and fever (n=21) (Table 1).

**Figure 2:**
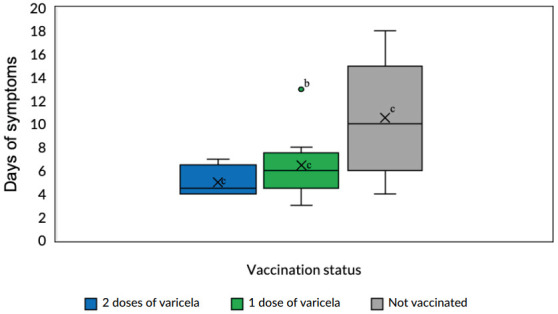
Symptom duration (in days) for varicella cases in relation to the varicella vaccination status. Itamaraju Indigenous Health Base, Bahia, 2024 (n=22)^a^

^a^Analysis included 22 cases with available information; ^b^Point outside the box of the group “1 dose of varicella” indicates outlier; ^c^“X” inside the boxes indicates the mean.

In 27 cases, it was possible to calculate the duration of symptoms, with a median of seven days (Min=3 and Max=18). When evaluating the duration of symptoms and number of doses received, information available in 22 cases, the median duration of symptoms was ten days for unvaccinated cases, six days for cases with one dose and four and a half days for people vaccinated with two doses (Figure 2).

**Figure 3:**
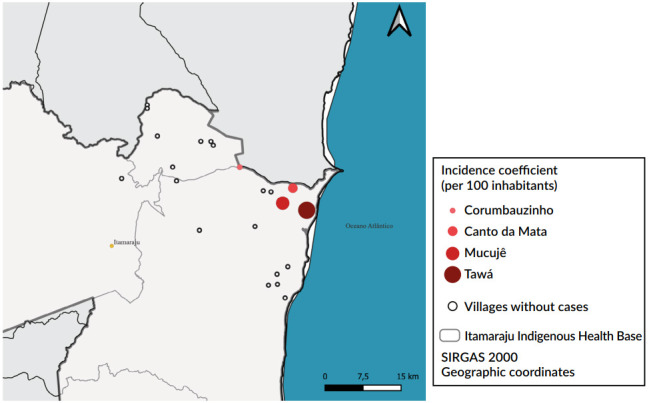
Representation of the incidence of varicella cases in the villages. Itamaraju Base, Bahia, 2024 (n=23)^a^

^a^The size of the circles is proportional to the incidence coefficient.

According to the cluster of cases in indigenous people (n=27) and cases in schoolchildren and education professionals (n=28), the occurrence of the outbreak was confirmed. The spatial distribution analysis of cases of residents in the Itamaraju Base Center villages (Figure 3) showed recorded cases in four of the 22 villages, located in the northeastern portion of the Base Center. The villages and their respective incidence coefficients were: Tawá (3.9 cases/100 inhabitants), followed by Mucujê (2.8 cases/100 inhabitants), Canto da Mata (2.1 cases/100 inhabitants) and Corumbauzinho (0.6 cases/100 inhabitants).

Most cases (n=28) occurred in the school setting. The Tawá Indigenous State School had the highest number of cases (n=25), with 21 students and four school professionals, with an attack rate of 6.2% (25/403). In addition to the Tawá school, there were three cases in students from the Mucujê village school, with an attack rate of 7.0% (3/43).

**Figure 4:**
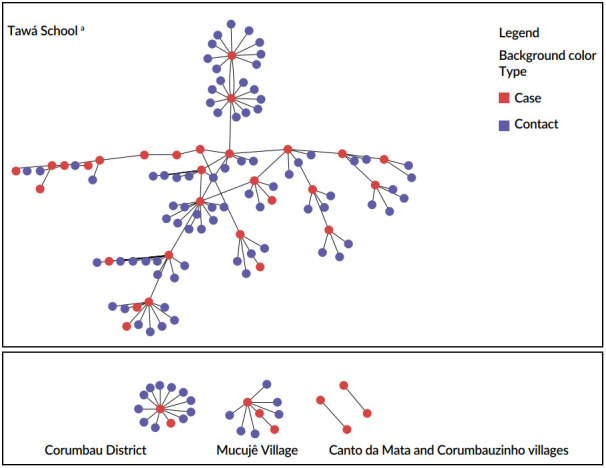
Transmission chains for the varicella cases and contacts investigated, Itamaraju Base, Bahia, 2024 (n=158; 42 cases and 116 contacts)^b^

^a^Tawá School (n=114; 30 cases and 84 contacts); Mucujê Village (n=10; 3 cases and 6 contacts); Canto da Mata Village (n=2; 2 cases); Corumbauzinho Village (n=2; 2 cases); Corumbau District (n=13; 2 cases and 11 contacts); ^b^Three cases and 15 contacts not represented in the scheme.

In total, 116 contacts were identified from 39 confirmed cases, a mean of three contacts for each case (Min=1 and Max=13). Ninety-one (78.4%) contacts were associated to cases by households, followed by associations by neighborhood (n=5; 4.3%), school transportation (n=4; 3.4%), and school/daycare center (n=1; 0.9). For 15 (12.9%) contacts, the type of association was not declared. The monitoring of contacts identified that six (5.2%) became cases. Five transmission chains were detected in the Tawá village with 29 cases, including students and family members, and chains in the Mucujê, Corumbauzinho, and Canto da Mata villages and the Corumbau district (one chain with 3 cases and three chains with two cases) (Figure 4).

Due to the diversity of dermatological diseases at the site, 17 biological samples were collected from seven patients in order to investigate varicella, Oropouche and Mayaro fever. Of the seven patients, one had laboratory confirmation, two were confirmed by clinical-epidemiological criteria and four were ruled out for varicella. There was no detection of the arboviruses researched. It should be noted that of the 17 samples, only six were collected in a timely manner for serology or molecular biology techniques. Two samples had hemolysis at collection.

As for the varicella vaccination coverage for the children participating in the survey (N=73), the 1st-dose coverage for eligible children was 78.3% (47/60) before the outbreak, rising to 90.0% (54/60) after blockade and intensification. The 2nd-dose coverage was 45.9% (17/37) and 70.3% (26/37), before and after the beginning of the outbreak, respectively.

The initiatives implemented by the territory’s health care teams included vaccination blockade in case contacts and vaccination intensification in the Tawá, Craveiro, Mucujê and Corumbauzinho villages, and - from May 23, 2024 to July 3, 2024 - 273 doses of monovalent varicella vaccine were administered in the indigenous and non-indigenous population, corresponding to 30 blockade doses and ten doses of tetraviral vaccine, with use of immunoglobulin in a pregnant patient.

## Discussion

As far as it was possible to determine, this is the first documented report of an investigation of a varicella outbreak in the indigenous population of the Pataxó ethnic group, located in the Itamaraju Base Center, Bahia. Most cases occurred in indigenous children, of both sexes, with school association, followed by household transmission.

As biases and limitations of this study, case selection bias may have occurred, in view of the possibility of individuals not notified by the local surveillance. To minimize it, we actively searched for cases. Another bias that may have occurred was information bias by self-declaration, since the study location is a region with intense interaction between indigenous and non-indigenous populations. One limitation is the low level of evidence of “case series” studies.

Most cases being in school-age children was expected due to the higher incidence in children^10^. The attack rate in the schools and the maintenance of the outbreak for about 14 weeks, similarly to a school in the city of Dongguan, China, may be explained by the vaccination coverage below adequate and the decreased effectiveness of the vaccine over time^11^.

The number of contacts of a varicella case varies greatly depending on the transmission setting (household, school or hospital), and studies have shown that a varicella case usually has one to two close household contacts, also depending on household size; however, in institutional or community settings, the number of contacts may be higher^12^.

This investigation found cases in vaccinated and unvaccinated individuals, which has been reported in other investigations^11^ ^), (^ ^13^ ^), (^ ^14^ ^), (^ ^15^, and it is essential that high vaccination coverage of the two doses is maintained to prevent the spread of the disease. The duration of symptoms in vaccinated individuals was shorter than in non-vaccinated individuals, showing that, despite the occurrence of infection, the disease was milder and with rapid evolution in vaccinated individuals^14^ ^), (^ ^16^. The lack of severe cases is consistent with the expected course of the disease and with the timely adoption of prophylactic measures, such as the administration of immunoglobulin^17^ ^), (^ ^18^.

Studies reporting analyses of vaccination coverage in indigenous populations are scarce. Some of these studies, specifically on the COVID-19 vaccine, found that, despite the indigenous population being a priority group for vaccination^19^, they had lower vaccination coverage compared to the general population^20^; this result may be reflected in the coverage of other vaccines, such as the varicella vaccine. Corroborating this finding, data indicate that vaccination coverage in the municipality of Prado in 2024 (80.47%)^21^ was higher than the coverage found in the Tawá village^21^. Indigenous populations may have lower vaccination coverage due to geographic isolation, lack of coordinated public health care strategies, misinformation and limited access to health care services. Addressing such disparities requires public health care interventions geared toward this population with culturally-sensitive communication^19^ ^), (^ ^20^.

This investigation enabled characterizing the clinical-epidemiological profile of varicella in this indigenous population, the vaccination status and verifying the existence of the outbreak, which ended with the preventive measures recommended by the research team and implemented by the local health care managers and providers.

## Data Availability

The databases used in this study are publicly accessible, and the links to obtain them are detailed in the methods. The tabulated data are available at: https://demo.dataverse.org/dataset.xhtml?persistentId=doi%3A10.70122%2FFK2%2FEMAN5Y&version=DRAFT.
