# Supplementary material for: Varicella outbreak at the Itamaraju Indigenous Health Base Center: a descriptive study, Bahia, 2024
Source: Epidemiol Serv Saude. 2026 Feb 13;35:e20250856. doi: 10.1590/S2237-96222026v35e20250856.en (PMC12904630; doi:10.1590/S2237-96222026v35e20250856.en)
Supplement: Translation

# Translation: Surto de varicela no Polo Base de Saúde Indígena de Itamaraju: estudo descritivo, Bahia, 2024

**DOI:** 10.1590/S2237-96222026v35e20250856.pt

**Authors:** Guilherme Alves de Siqueira, Leticia Stanczyk, Benvindo Joãozinho Sá, Lucimeire Neris Sevilha da Silva Campos, Vanessa Cristina Fragoso Farias, Pedro Ricardo da Silva Biscarde, Ana Carolina de Castro Silva, Aluciena Dias da Silva Dias, Sonia Helena Afonso, Adriana Dourado de Carvalho, Rosidalva Barreto da Silva Sousa, Tatiane Ferreira dos Santos, Solange Nunes Souza Monte, Octavia Lorena P. Falcao, Samara Carolina Rodrigues, Aroldo José Borges Carneiro

**Affiliations:** 1 Ministério da Saúde, Secretaria de Vigilância em Saúde e Ambiente, Brasília, DF, Brasil; 2 Distrito Sanitário Especial Indígena da Bahia, Salvador, BA, Brasil; 3 Distrito Sanitário Especial Indígena da Bahia, Itamaraju, BA, Brasil; 4 Secretaria da Saúde do Estado da Bahia, Salvador, BA, Brasil; 5 Secretaria da Saúde do Estado da Bahia, Teixeira de Freitas, BA, Brasil; 6 Secretaria Municipal de Saúde de Prado, Prado, BA, Brasil

**Keywords:** Varicela, Surtos de Doenças, Saúde de Populações Indígenas, Vacinação, Estudos Descritivos

## Abstract

**Objetivo:**

Investigar casos de varicela no território indígena do Polo de Itamaraju e calcular cobertura vacinal contra varicela na aldeia Tawá, Bahia, 2024.

**Métodos:**

Estudo descritivo de série de casos e inquérito vacinal censitário em crianças da aldeia Tawá, realizados entre 6 de junho e 3 de julho de 2024, sobre banco de dados secundários da investigação, disponibilizado pela Coordenação-Geral de Doenças Imunopreveníveis. Utilizaram-se estatística descritiva com medidas de frequência absoluta e relativa, e tendência central e dispersão.

**Resultados:**

Identificamos 42 casos, igualmente distribuídos entre os sexos, 27 eram indígenas e 19 crianças de sete a 13 anos, 14 casos não foram vacinados contra varicela. Vinte e três ocorreram nas aldeias Tawá, Mucujê, Canto da Mata e Corumbauzinho, e 25 vinculados a uma escola da aldeia Tawá, onde estudavam indígenas e não indígenas, com taxa de ataque de 6,2% (25/403). Em outra escola indígena (aldeia Mucujê) a taxa de ataque foi 7,0% (3/43). Sintomas dos casos com informação disponível (n=31) mais frequentes foram exantema (31/31), prurido (24/31) e febre (21/31). Cobertura vacinal contra varicela foi 1ª dose 90,0% (54/60) e 2ª dose 70,3% (26/37). Dezesseis crianças receberam vacina contra varicela após início do surto, sete receberam a primeira dose e nove a segunda. Uma gestante utilizou imunoglobulina. O surto foi controlado 101 dias após primeiro caso.

**Conclusão:**

Confirmou-se a ocorrência do surto no território indígena. Situação vacinal contra varicela na aldeia Tawá estava abaixo da meta preconizada pelo Programa Nacional de Imunizações. Implementou-se atualização vacinal e busca ativa de casos.

**Aspectos éticos**

**Esta pesquisa respeitou os princípios éticos, obtendo os seguintes dados de aprovação:**

Comitê de ética em pesquisa: Comissão Nacional de Ética em Pesquisa

Número do parecer: 7.640.452

Data de aprovação: 16/6/2025

Certificado de apresentação de apreciação ética: 88056825.4.0000.0008

Registro do consentimento livre e esclarecido: Dispensado pelo comitê de ética em pesquisa.

## Introdução

Varicela é uma doença viral, febril, aguda e altamente contagiosa descrita pelo aparecimento de exantema maculopapular de distribuição centrípeta (centro para extremidades), que se torna vesicular, evoluindo para pústulas e crostas secas não infecciosas, num período de três a sete dias^1^. O principal sintoma da doença é o polimorfismo das lesões cutâneas, que podem apresentar as diversas formas evolutivas em uma mesma região do corpo, acompanhadas de prurido^2^. A evolução em crianças é usualmente não grave e autolimitada. Em adolescentes e adultos, geralmente, a clínica é mais exuberante. Já infecções subclínicas são raras^1^. Há escassez de relatos de surtos de varicela em populações indígenas no Brasil, o que dificulta parâmetros operacionais para controle.

O agente etiológico é o vírus varicela-zoster, família Herpesviridae, subfamília Alphaherpesvirinae, genoma de tipo ácido desoxirribonucleico, tendo como reservatório o ser humano. Após primeira infecção o vírus permanece em latência no sistema nervoso, podendo ser reativado na idade adulta, sendo conhecida como herpes-zóster^1^. É transmitida: pessoa a pessoa (contato direto), através de secreções respiratórias (aerossóis) ou pelo exsudato das lesões (menos frequente); e de forma indireta, por meio de objetos contaminados^1^.

O período de incubação da doença é de dez a 21 dias após contato com caso de varicela. O período de transmissibilidade tem início dois dias antes do início do exantema e dura até que todas as lesões estejam na fase de crosta.

A prevenção da doença acontece por meio de vacina, que possui licença no Brasil nas apresentações monovalente ou tetraviral (componentes sarampo, caxumba, rubéola e varicela), disponibilizada pelos serviços públicos de saúde na sua rotina com esquema de duas doses, uma aos 15 meses e outra aos 4 anos. Crianças não vacinadas de forma oportuna podem receber a vacina monovalente até 6 anos, 11 meses e 29 dias^3^. A imunoglobulina humana antivaricela é recomendada como medida profilática para grupos específicos em que existe contraindicação de uso da vacina como: crianças menores de nove meses, gestantes e pessoas imunodeprimidas.

Em situações de surto, a vacinação deve acontecer de forma seletiva e conforme indicado no Calendário Nacional de Vacinação^3^, que preconiza o período oportuno de 120 horas (cinco dias) para administração da vacina e 96 horas (quatro dias) para administração da imunoglobulina humana antivaricela, após contato com caso suspeito ou confirmado de varicela. Os surtos e os casos graves (internados ou óbitos) são de notificação compulsória nacional imediata. No entanto, no estado da Bahia a notificação de casos leves de varicela também é de notificação compulsória, por meio de preenchimento da ficha de notificação individual do Sistema de Informação de Agravos de Notificação.

O diagnóstico clínico é o principal critério de confirmação de caso, considerando a sintomatologia. O diagnóstico laboratorial não é utilizado na rotina da rede de Laboratórios de Saúde Pública, exceto no diagnóstico diferencial de casos graves e óbitos, ou em apresentações clínicas menos típicas. O diagnóstico diferencial pode ser feito para outras infecções cutâneas, como: varíola (erradicada); coxsackioses; dermatite herpetiforme; impetigo; erupção variceliforme de Kaposi; riquetsioses, entre outras.

Em buscas na literatura não foram identificados relatos de surtos de varicela em população indígena. Todavia, existe o registro de surto de varicela em creches e escolas, em 2005, no estado de São Paulo, com 118 casos, uma hospitalização e nenhum óbito^4^ e outro surto entre imigrantes venezuelanos alojados em abrigos no estado de Roraima, em 2019, quando foram detectados 38 casos ativos, um caso apresentando complicações, sendo adotadas ações de imunização para controle da transmissão^5^.

No dia 10/3/2024, um paciente indígena, de 11 anos, proveniente da aldeia Tawá, Polo Base de Itamaraju, foi atendido pela Equipe Multidisciplinar de Saúde Indígena apresentado febre, indisposição e presença de uma vesícula no corpo, recebendo diagnóstico de impetigo bolhoso. Em 13/4/2024 com piora do quadro clínico, o paciente foi levado pelos responsáveis para atendimento médico na Unidade de Pronto Atendimento de Prado, havendo a confirmação diagnóstica do primeiro caso de varicela na aldeia Tawá.

Em 14/5/2024, sete casos haviam sido notificados, incluindo uma professora da escola indígena onde estudava o primeiro caso, sendo esta frequentada por indígenas e não indígenas. No dia 17/5/2024, a Equipe Multidisciplinar de Saúde Indígena comunicou o surto ao Distrito Sanitário Especial Indígena da Bahia. Naquela data a situação vacinal contra varicela da aldeia Tawá era desconhecida.

Em 29/5/2024, o Distrito Sanitário Especial Indígena da Bahia convidou o Programa de Treinamento em Epidemiologia Aplicada aos Serviços do Sistema Único de Saúde (EpiSUS-Avançado), juntamente com a Coordenação-Geral de Vigilância das Doenças Imunopreveníveis, para apoiar a investigação de casos de varicela na aldeia Tawá.

No dia 6/6/2024, a equipe do EpiSUS-Avançado e área técnica da Coordenação-Geral de Vigilância das Doenças Imunopreveníveis se deslocou para o território, com a reunião de chegada acontecendo no dia seguinte. Em 10/6/2024, diante da possibilidade de ocorrência de mais casos em outras aldeias do Polo Base de Itamaraju, o Distrito Sanitário Especial Indígena da Bahia solicitou a expansão do local de investigação para toda abrangência do Polo.

Os objetivos do estudo foram investigar os casos de varicela em território indígena do Polo de Itamaraju e calcular cobertura vacinal contra varicela na aldeia Tawá, Bahia, 2024.

## Métodos

### Delineamento

Trata-se de um estudo composto por dois itens: (i) descrição dos casos de varicela (série de casos) e (ii) inquérito vacinal censitário em crianças da aldeia Tawá, Polo Base de Itamaraju, extremo sul do estado da Bahia, Nordeste, Brasil.

### Contexto

O Polo Base de Itamaraju, segundo dados demográficos do Distrito Sanitário Especial Indígena da Bahia, possuía, em maio de 2024, 3.750 indígenas das etnias Pataxó e Pataxó Hã Hã Hãe, divididos em 22 aldeias distribuídas nos municípios de Itamaraju, Prado, Alcobaça e uma porção de Porto Seguro^6^. Entre as aldeias, destacam-se a Tawá (410 habitantes), Corumbauzinho (360 habitantes), Mucujê (108 habitantes) e Canto da Mata (95 habitantes). Essa população indígena tem contato com não indígenas desde o século XVI e são falantes da língua portuguesa. É uma região de intenso fluxo turístico, em especial o distrito de Corumbau, em Prado^7^ ^), (^ ^8^.

### Participantes

Para a série de casos, a população do estudo foi constituída pelos casos notificados de varicela entre 1 de janeiro de 2024 e 3 de julho de 2024, com os respectivos contatos, identificados por meio de entrevistas com os casos ou seus responsáveis, na área adstrita do Polo Base de Saúde Indígena de Itamaraju. Foram adotadas as seguintes definições:

- Caso suspeito de varicela: indivíduo residente nos municípios da área de abrangência do Polo Base de Itamaraju, Bahia que, de 1/1/2024 a 3/7/2024, tenha sido notificado após apresentar quadro discreto de febre moderada, de início súbito, que durou de dois a três dias, e sintomas generalizados inespecíficos (mal-estar, adinamia, anorexia, cefaleia e outros) e erupção cutânea pápulo-vesicular, que se inicia na face, no couro cabelo ou no tronco (distribuição centrípeta-cabeça e tronco);
- Caso confirmado de varicela: caso suspeito com exantema maculopapulovesicular difuso cujas vesículas evoluíram para crostas, em dois a três dias, sem outra causa aparente, com ou sem confirmação laboratorial;
- Caso descartado de varicela: caso suspeito cuja avaliação clínico-epidemiológica conclua como sendo outra doença;
- Contato: indivíduo que teve contato com um caso confirmado no período de dois dias antes do aparecimento do exantema até todas as lesões virarem crostas;
- Surto de varicela: ocorrência de casos agregados em creches, escolas, instituições de longa permanência, população privada de liberdade, áreas indígenas, entre outras^9^, surtos de varicela são considerados controlados após 21 dias sem novos casos.

Para o inquérito, a população foi formada pelos responsáveis por indígenas com idade inferior a 7 anos, residentes na aldeia Tawá, entre 6/6/2024 e 4/7/2024.

### Variáveis de interesse

As variáveis de interesse referentes aos casos incluíram características sociodemográficas (sexo, idade em anos, raça/cor da pele e local de residência) e clínico-epidemiológicas (classificação do caso em confirmado ou descartado, evolução clínica, critério de confirmação, sintomas apresentados, datas de início dos sintomas, de notificação e de evolução clínica, histórico vacinal, histórico de exposição e local provável de infecção). Para os contatos foram consideradas as mesmas variáveis sociodemográficas, e clínico-epidemiológicas (datas de primeiro e último contato com caso), natureza da exposição (familiar, vínculo de trabalho, estudo) e apresentação de sinais ou sintomas nos últimos 30 dias (Sim ou Não). As variáveis de interesse referentes às crianças participantes do inquérito foi a verificação de doses aplicadas preconizadas para cada idade (respondente era questionado caso houvesse doses em atraso).

### Fontes de dados

Dados secundários anonimizados disponibilizados pela Coordenação-Geral de Vigilância das Doenças Imunopreveníveis, gerados pelo Programa de Treinamento em Epidemiologia Aplicada aos Serviços do Sistema Único de Saúde (EpiSUS-Avançado), durante investigação de surto de varicela. Os dados de notificação foram obtidos nas fichas do Sistema de Informação de Agravos de Notificação. Dados cartográficos foram disponibilizados pelo Distrito Sanitário Especial Indígena e dados públicos do Instituto Brasileiro de Geografia e Estatística.

### Viés

Podem ter ocorrido vieses de seleção, pela possibilidade de subnotificação de casos, e de informação, pela autodeclaração. Esses vieses foram minimizados por meio de busca ativa e checagem dos registros.

### Tamanho do estudo

Série de casos de 42 notificações confirmadas para varicela no Sistema de Informação de Agravos de Notificação, oriundas do Polo Base de Saúde Indígena Itamaraju. E inquérito vacinal censitário com 73 crianças residentes na aldeia Tawá.

### Pesquisa laboratorial

Foi realizada sorologia para pesquisa de anticorpos contra varicela e transcrição reversa seguida de reação em cadeia da polimerase (RT-qPCR) para pesquisa das arboviroses febre do Oropouche e Mayaro, de forma complementar e não utilizado como critério de inclusão, apenas como diagnóstico confirmatório ou diferencial.

As ações de prevenção e controle implementadas pelas equipes de investigação do surto contaram com apoio de equipes de saúde indígena, equipes municipais e estaduais de imunização, com a administração de imunoglobulina em gestantes e realização de bloqueio e intensificação vacinal, com as vacinas tetraviral ou monovalente, de acordo com o preconizado pelo Programa Nacional de Imunizações.

### Análise estatística

A análise dos dados se deu por meio de estatística descritiva, com medidas de frequência absolutas e relativas e medidas de tendência central e dispersão. Para os casos em escolas, onde se tinha conhecimento da população exposta, foi realizado o cálculo de taxa de ataque por meio da fórmula:

$$\mathrm{Taxa de ataque}=\frac{\mathrm{Casos confirmados de varicela}}{\mathrm{Pessoas expostas}}*100\%$$

Também foi calculado o coeficiente de incidência para cada aldeia com registros de casos da seguinte forma:

$$\mathrm{Coeficiente de incidência}=\frac{\mathrm{Casos confirmados de varicela}}{\mathrm{População da aldeia}}*100\;\mathrm{habitantes}$$

O coeficiente de incidência foi utilizado representativamente para construção do mapa temático.

O cálculo da cobertura vacinal contra varicela, se deu pela seguinte fórmula:

$$\mathrm{Cobertura vacinal}=\frac{\mathrm{Dose aplicada do imunobiológico em crianças}}{\mathrm{Crianças elegíveis por imunobiológico}}*100$$

Foram utilizados os programas Go. Data 2.49.0, Microsoft Excel 2013, R.4.3.1 e R. Studio, QGIS 3.30.2 e o Research Eletronic Data Capture (REDCap) 10.6.0.

## Resultados

Foram notificados 57 casos suspeitos de varicela, sendo 42 confirmados e 15 descartados. Destes, cinco foram diagnosticados com alergia, quatro com impetigo, quatro não evoluíram com clínica de varicela, um com dermatite e um com síndrome mão-pé-boca. Não foram registrados óbitos ou casos graves de varicela. O inquérito vacinal abrangeu 73 crianças, contemplando as 69 indígenas registradas pelo censo do Distrito Sanitário Especial Indígena em maio de 2024, acrescendo quatro por mudança. Foram entrevistados 61 responsáveis e todas as crianças possuíam cartão de vacina.

O primeiro caso notificado teve início de sintomas em 9/4/2024. Com a busca ativa, intensificação vacinal e aumento da sensibilidade dos profissionais, houve aumento das notificações, com pico de registros em 13/5/2024 (Figura 1). O último caso registrado teve data de início dos sintomas em 28/6/2024. Em 19/7/2024, após 21 dias sem caso novo e 101 dias desde o primeiro caso, o surto foi considerado controlado (Figura 1).

**Figura 1:** Casos confirmados de varicela, segundo data de início dos sintomas. Polo Base de Saúde Indígena de Itamaraju, Bahia, 2024 (n=42)

Nos 42 casos, houve distribuição igualitária entre os sexos, 27 se autodeclararam indígenas, 13 pardos, uma branca e uma preta (Tabela 1). Vinte e três pessoas informaram morar em território indígena, sendo a maioria na aldeia Tawá (n=16), seguida pela aldeia Mucujê (n=3), e as aldeias Corumbauzinho e Canto da Mata, com dois casos cada. Dos casos em território não indígena, 18 residiam em Corumbau, um distrito do município de Prado a 17 quilômetros de distância da aldeia Tawá. Quando questionados acerca do local provável de infecção, dos 27 que conseguiram determinar, 13 relataram como local provável de infecção a escola, 12 a residência e dois a vizinhança (Tabela 1).

**Tabela 1:** Características sociodemográficas e epidemiológicas dos casos confirmados de varicela. Polo Base de Saúde Indígena de Itamaraju, Bahia, 2024 (n=42)

Variável	n
**Sexo**	
Masculino	21
Feminino	21
**Raça/cor da pele**	
Indígena	27
Parda	13
Branca	1
Preta	1
**Local de residência**	
Corumbau	18
Aldeia Tawá	16
Aldeia Mucujê	3
Aldeia Canto da Mata	2
Aldeia Corumbauzinho	2
Palmares	1
**Local provável de infecção**	
Escola	13
Residência	12
Vizinhança	2
Não soube determinar	15
**Faixa etária (anos)**	
1-7	13
8-14	19
15-21	5
22-28	1
29-35	2
>35	2
**Vacina contra varicela**	
Não	14
1 dose	13
2 doses	9
Sem informação	6
**Sinais e sintomas (n=31)** ^a^	**n (%)**
Exantema	31 (100,0 %)
Prurido	24 (77,4 %)
Febre	21 (67,7 %)
Cefaleia	18 (58,1 %)
Falta de apetite	13 (41,9 %)
Irritabilidade	8 (25,8 %)
Vômito	3 (9,6 %)

^a^Informações sobre sinais e sintomas disponíveis apenas para 31 dos 42 casos.

As faixas etárias mais acometidas foram de 8 a 14 anos (n=19) e um a 7 anos (n=13). Acerca da situação vacinal contra a varicela, 14 dos casos não eram vacinados, seguidos por 13 pessoas com uma dose e nove pessoas com duas doses. Seis pessoas não possuíam informação de registro vacinal. Os sinais e sintomas apresentados incluíram exantema (n=31), prurido (n=24) e febre (n=21) (Tabela 1).

Em 27 casos foi possível calcular a duração dos sintomas, sendo a mediana de sete dias (Mín=3 e Máx=18). Quando avaliada a duração dos sintomas e número de doses recebidas, informação disponível em 22 casos, a mediana de duração dos sintomas foi de dez dias para casos não vacinados, seis dias para casos com uma dose e quatro dias e meio para pessoas vacinadas com duas doses (Figura 2).

**Figura 2:** Duração dos sintomas (em dias) dos casos de varicela em relação ao status da vacinação contra a doença. Polo Base de Saúde Indígena de Itamaraju, Bahia, 2024 (n=22)^a^

^a^Análise incluiu 22 casos com informação disponível; ^b^Ponto fora da caixa do grupo “1 dose de varicela” indica outlier; ^c^“X” dentro das caixas indica a média.

Pelo agregado de casos em indígenas (n=27) e casos em escolares e profissionais da educação (n=28), se confirmou a ocorrência do surto. A análise da distribuição espacial dos casos de residentes nas aldeias do Polo Base de Itamaraju (Figura 3), apontou registro de casos em quatro das 22 aldeias, localizadas na porção nordeste do Polo Base. As aldeias e os seus respectivos coeficientes de incidência foram: Tawá (3,9 casos/100 habitantes), seguida das aldeias Mucujê (2,8 casos/100 habitantes), Canto da Mata (2,1 casos/100 habitantes) e Corumbauzinho (0,6 casos/100 habitantes).

**Figura 3:** Representação da incidência de casos de varicela das aldeias. Polo Base de Itamaraju, Bahia, 2024 (n=23)^a^

^a^O tamanho dos círculos é proporcional ao coeficiente de incidência.

A maior parte dos casos (n=28) ocorreu no âmbito escolar. O Colégio Estadual Indígena Tawá teve o maior número de casos (n=25), sendo 21 estudantes e quatro profissionais da escola, com taxa de ataque de 6,2% (25/403). Além do Colégio Tawá, houve três casos em estudantes do Colégio da aldeia Mucujê, com taxa de ataque de 7,0% (3/43).

Foram identificados 116 contatos de 39 casos confirmados, uma média de três contatos para cada caso (Mín=1 e Máx=13). Noventa e um (78,4%) contatos estavam vinculados aos casos por agregados familiares, seguidos por vínculos de vizinhança (n=5; 4,3%), transporte escolar (n=4; 3,4%), e escolar/creche (n=1; 0,9). Para 15 (12,9%) contatos o tipo de vínculo não foi declarado. O monitoramento dos contatos identificou que seis (5,2%) se tornaram casos. Foram detectadas cinco cadeias de transmissão na aldeia Tawá com 29 casos, dentre alunos e familiares, e cadeias nas aldeias Mucujê, Corumbauzinho, Canto da Mata e do distrito de Corumbau (uma cadeia com três casos e três cadeias com dois casos) (Figura 4).

**Figura 4:** Cadeias de transmissão dos casos de varicela e contatos investigados, Polo Base de Itamaraju, Bahia, 2024 (n=158; 42 casos e 116 contatos)^b^

^a^Colégio Tawá (n=114; 30 casos e 84 contatos); Aldeia Mucujê (n=10; 3 casos e 6 contatos); Aldeia Canto da Mata (n=2; 2 casos); Aldeia Corumbauzinho (n=2; 2 casos); Distrito de Corumbau (n=13; 2 casos e 11 contatos); ^b^Três casos e 15 contatos não representados no esquema.

Devido a diversidade de doenças dermatológicas no local, foi realizada a coleta de 17 amostras biológicas referentes a sete pacientes, a fim de pesquisa de varicela, febre do Oropouche e Mayaro. Dos sete pacientes, um teve confirmação laboratorial, dois foram confirmados pelo critério clínico-epidemiológico e quatro foram descartados para varicela. Não houve detecção das arboviroses pesquisadas. Cabe destacar que das 17 amostras, apenas seis foram coletadas de forma oportuna para as técnicas de sorologia ou biologia molecular. Duas amostras tiveram hemólise na coleta.

Em relação à cobertura vacinal contra varicela das crianças participantes do inquérito (N=73), a cobertura da 1ª dose para as crianças elegíveis era de 78,3% (47/60) antes do surto, passando a 90,0% (54/60) após bloqueio e intensificação. Para a 2ª dose, a cobertura foi de 45,9% (17/37) e 70,3% (26/37), antes e após o início do surto, respectivamente.

Entre as ações realizadas pelas equipes de saúde do território, procedeu-se o bloqueio vacinal em contatos dos casos e intensificação vacinal nas aldeias Tawá, Craveiro, Mucujê e Corumbauzinho, sendo aplicadas, no período de 23/5/2024 a 3/7/2024, 273 doses de varicela monovalente na população indígena e não indígena, correspondendo a 30 doses de bloqueio e dez de vacina tetraviral, com utilização de imunoglobulina em uma paciente gestante.

## Discussão

Até onde foi possível determinar, este é o primeiro relato documentado de uma investigação de surto de varicela na população indígena da etnia Pataxó, localizada no Polo Base de Itamaraju, Bahia. A maioria dos casos aconteceu em crianças indígenas, de ambos os sexos, com vínculo escolar, seguido de transmissão domiciliar.

Como vieses e limitações deste estudo, podem ter ocorrido viés de seleção de casos, tendo em vista a possibilidade de indivíduos não notificados pela vigilância local. Para minimizar, realizamos busca ativa de casos. Outro viés que pode ter ocorrido foi de informação pela autodeclaração, já que o local do estudo é uma região de intensa interação entre população indígena e não indígena. Uma limitação é o baixo nível de evidência de estudos do tipo “série de casos”.

A maior parte dos casos em crianças em idade escolar era algo esperado devido a maior incidência ser no público infantil^10^. A taxa de ataque nas escolas e a manutenção do surto por cerca de 14 semanas, à semelhança do observado em uma escola na cidade de Dongguan, na China, podem ter explicação na cobertura de vacinação abaixo do adequado e na diminuição da eficácia da vacina com o tempo^11^.

O número de contatos de um caso de varicela tem uma grande variação a depender do ambiente de transmissão (domicílio, escolas ou hospitais), e estudos mostraram que um caso de varicela geralmente tem de um a dois contatos domiciliares próximos, a depender também do tamanho da família, contudo em ambientes institucionais ou comunitários o número de contatos pode ser maior^12^.

Nesta investigação houve casos em indivíduos vacinados e não vacinados, algo já relatado em outras investigações^11^ ^), (^ ^13^ ^), (^ ^14^ ^), (^ ^15^, sendo fundamental que altas coberturas vacinais das duas doses sejam mantidas para impedir a disseminação da doença. A duração dos sintomas em indivíduos vacinados foi menor do que em não vacinados, mostrando que, apesar de ocorrer a infecção, a doença aconteceu de forma mais branda e de rápida evolução nos vacinados^14^ ^), (^ ^16^. A ausência de casos graves é compatível com o curso esperado da doença e com a adoção oportuna de medidas profiláticas, como a administração de imunoglobulina^17^ ^), (^ ^18^.

Poucos estudos reportam análises de cobertura de vacinação em populações indígenas. Alguns destes, realizados especificamente sobre a vacina contra a covid-19, verificaram que, apesar da população indígena ser um grupo prioritário para a vacinação^19^, estes possuíam menor cobertura vacinal comparados à população geral^20^, resultado que pode refletir nas coberturas de outras vacinas, a exemplo da varicela. Corroborando esse achado, dados apontam que a cobertura vacinal no munícipio de Prado em 2024 (80,47%)^21^ foi superior à cobertura encontrada na aldeia Tawá^21^. As razões para a população indígena apresentar coberturas vacinais mais baixas podem se dar pelo isolamento geográfico, pela falta de estratégias coordenadas de saúde pública, pela desinformação e pelo acesso limitado à saúde. Lidar com tais disparidades requer intervenções de saúde pública direcionadas a esta população com comunicação culturalmente sensível^19^ ^), (^ ^20^.

A partir desta investigação foi possível caracterizar o perfil clínico-epidemiológico da varicela nessa população indígena, a situação vacinal e comprovar a existência do surto, que se encerrou com as medidas de prevenção recomendadas pela equipe de investigação e implementadas pelos gestores e profissionais da saúde local.

## Data Availability

As bases de dados utilizadas neste estudo são de acesso público, e os links para sua obtenção estão detalhados nos métodos. Os dados tabulados estão disponíveis em: https://demo.dataverse.org/dataset.xhtml?persistentId=doi%3A10.70122%2FFK2%2FEMAN5Y&version=DRAFT.
